# Synthesis and Calcium Mobilization Activity of cADPR Analogues Which Integrate Nucleobase, Northern and Southern Ribose Modifications 

**DOI:** 10.3390/molecules17044343

**Published:** 2012-04-10

**Authors:** Yue Zhou, Peilin Yu, Hongwei Jin, Zhenjun Yang, Jianbo Yue, Liangren Zhang, Lihe Zhang

**Affiliations:** 1 State Key Laboratory of Natural and Biomimetic Drugs, School of Pharmaceutical Sciences, Peking University, Beijing 100191, China; 2 Department of Physiology, University of Hong Kong, Hong Kong, China

**Keywords:** cADPR analogue, nucleotide, synthesis, calcium mobilization

## Abstract

Novel cADPR mimics, which integrate nucleobase, northern and southern ribose modifications were synthesized. The key steps of the synthesis were a Cu(I)-catalyzed Hüisgen [3+2] cycloaddition and a microwave-assisted intramolecular pyrophosphorylation. Preliminary biological investigations showed that these cADPR mimics are membrane-permeating agonists of the calcium signaling pathway. The introduction of chlorine or fluorine at the 2'-position of the southern riboses led to a decrease of activity. The existence of a hydrophobic group on the 3'-OH of the southern riboses does not obviously alter the agonistic activity.

## 1. Introduction

**Figure 1:**
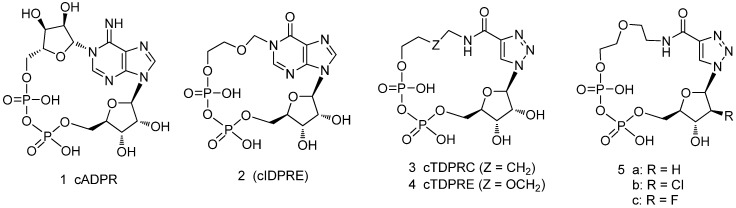
Structures of cADPR analogues.

Cyclic adenosine disphosphate ribose (cADPR, **1**, Figure 1) is a universal Ca^2+^ mobilizing secondary messenger first identified in the sea urchin egg system [1]. Since its discovery, numerous cell systems that utilize the cADPR/ryanodine receptor (RyR) Ca^2+^ signaling system to control Ca^2+^-dependent cellular responses, such as fertilization, secretion, contraction, proliferation and so on have been described [2,3]. A large number of proteins are involved in specifically shaping Ca^2+^ signals, however, it is still unclear exactly how cADPR elicits calcium release, which includes whether specific cADPR binding proteins exist. Since the discovery of cADPR, a number of structurally diverse cADPR analogues have been synthesized and used to elucidate the molecular mechanism of calcium signaling [4,5,6]. The syntheses could be divided into chemo-enzymatic or chemical synthesis [7,8]. The structures include modifications of the pyrophosphate [9,10,11,12], purine [13,14,15,16] and southern and northern riboses [17,18,19,20] of cADPR. These analogues can agonize or antagonize cADPR/RyR calcium signal pathway. 

cADPR analogues with modifications on the northern and southern riboses are the most investigated. Shuto *et al.* found that the effect of modification of the northern ribose on calcium signaling depended on the cell system [21,22]. Our previous studies showed that the northern ribose of cADPR tolerated structural modifications to some extent. The agonistic activity is mostly maintained even if the northern ribose is replaced by ether or alkane linkages, such as in cIDPRE (**2**), and this modification makes analogues cell-permeant [18]. Potter *et al.* investigated the importance of the 2'-OH on the calcium signaling behavior of 8-substituted cADPR derivatives. They found that the 2'-OH group does not affect the Ca^2+^-mobilizing ability of cADPR itself, but that it is an important motif for the antagonistic activities of 8-substituted cADPR analogues [23]. The C2'-*endo/syn* conformation is crucial for agonistic or antagonistic activity in sea urchin egg homogenates [24]. The findings implied the coordinating effect of nucleobase and riboses on the activity of cADPR analogues. 

The nucleobase of cADPR was simplified in our previous study; we have found that triazole-based cADPR analogues **3**,**4** are cell-permeating mild agonists of the cADPR/RyR calcium pathway [25]. To elucidate the structure-activity relationships of cADPR analgues in more detail, and provide probes for investigation of the molecular mechanism of cADPR regulated calcium pathways, we have designed and synthesized novel cADPR analogues which integrate three types of modifications of the nucleobase, northern and southern riboses (compounds **5**). In this study, the nucleobase is replaced by a simplified triazole moiety, the northern ribose is replaced by an ether linkage and the southern ribose is replaced by 2'-deoxy or 2'-deoxy-2'-haloribofuranoses, respectively. 

## 2. Results and Discussion

### 2.1. Chemistry

**Scheme 1:**
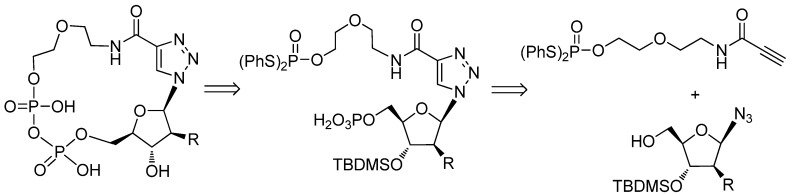
Retrosynthesis of cADPR analogues.

The synthesis could be generalized to three steps, *i.e.*, the synthesis of phosphorylated northern and southern moieties, the assembly of the triazole moiety and intramolecular cyclization (Scheme 1). The phosphorylation was performed before the coupling of the northern and southern moieties. Microwave-assisted intramolecular cyclization was used to form the pyrophosphates of the cADPR analogues.

**Scheme 2:**
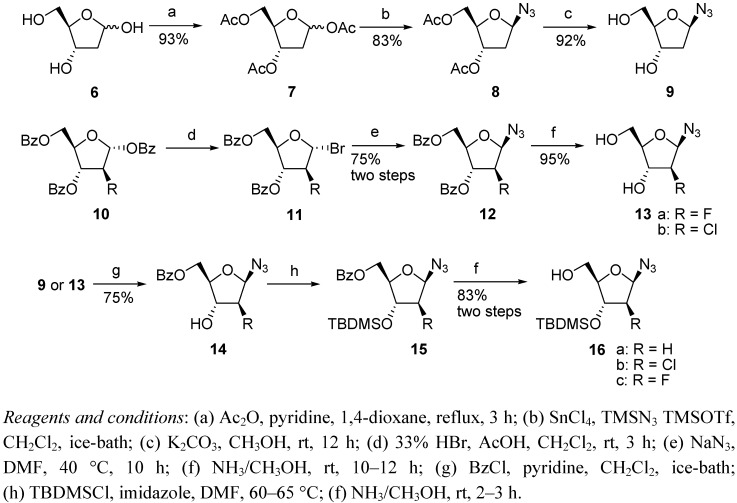
Synthesis of 1-azido-2-modified sugars.

For the syntheses of the southern moieties, various strategies were adopted to construct 1-azido-2-modified sugars **16**, which are summarized in Scheme 2.

1-β-D-Azido-2-deoxyribose (**9**) was prepared starting from 2-deoxyribose based on Greenberg’s method [26]. The azidation of triacetylribose **7** gave a pair of anomers. The α:β isomer ratio was 1:8 and they could be separated by silica gel column chromatography. The fluroarabinose **10a** was used directly from commercial material [27]. The chloroarabinose **10b** was synthesized by the triflation of commercial available 1,3,5-tri-*O*-benzoyl-α-D-ribofuranose, followed by treatment with LiCl in *N*-methylpyrrolidinone (NMP) [28]. Treatment of **10** with HBr/CH_3_COOH in dichloromethane followed by the addition of sodium azide in DMF at 30 °C for 12 h gave **12** in a yield of 75%. Considering the instability of the anomeric bromide, intermediate **11** was used without purification. The 3-OH groups of **9** and **13** were protected by *tert*-butyldimethyl silyl (TBDMS) groups by reaction with TBDMSCl after their 5-OH groups were selectively protected as benzoyl groups, thus giving **15**. After treatment with NH_3_/CH_3_OH, compound **16** was obtained.

**Scheme 3:**
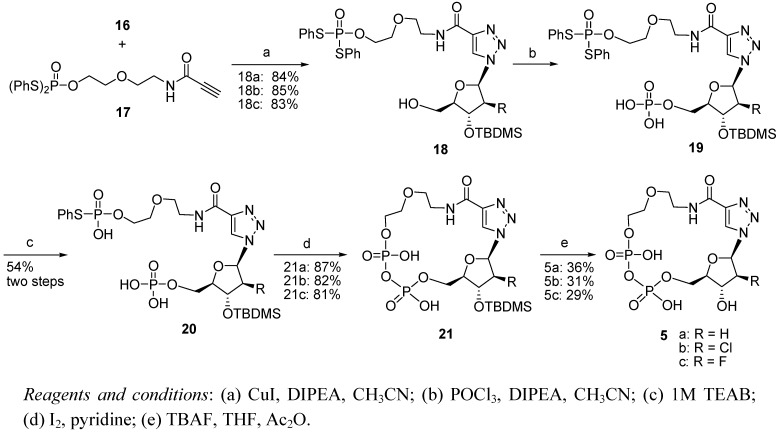
Synthesis of compound **5**.

Cu(I)-catalyzed Hüisgen [3+2] cycloaddition between terminal alkyne and azide groups was adopted to build the 1,2,3-triazole unit (Scheme 3). The terminal alkyne building block **17** which carried a *S*,*S*-diphenylphosphate group was prepared by the reported procedure [29]. Several classical Cu(I) catalysis systems were used to catalyze the Hüisgen [3+2] cycloaddition reaction of **16** and **17**. Under optimal conditions, compound **18** was obtained in a yield of 85%. Compound **18** was phosphorylated by using POCl_3_/DIPEA in CH_3_CN, followed with purification by HPLC eluting with 0.05M triethylammonium bicarbonate (TEAB, pH = 7.5). It was found that the *S*,*S*-diphenylphosphate group was sensitive to alkaline conditions and was decomposed to *S*-phenylphosphate. Compound **20** was obtained as a triethylammonium salt in a yield of 54% for two steps. 

Microwave-assisted organic chemistry has been recently developed for the efficient synthesis of functional compounds in many areas [30,31]. In most previous works, intermolecular reactions for the formation of pyrophosphates of cADPR analogues were performed in super-dilute solutions [8]. Under these super-dilute solution conditions, the reaction was usually completed by using a syringe-pump over 20 h. In addition, the reaction was very sensitive to traces of water. This strictly dry environment and the long reaction time in a super-dilute solution made the experimental work-up tedious, and thus, the large-scale preparation of cADPR analogues was difficult. Under microwave-assisted conditions, the overall efficiency of intramolecular pyrophosphorylation has been greatly improved [29]. We applied this technology to compound **20** to prepare **21**. Optimization of the reaction conditions was done by changing temperature and reaction time. The reaction of **20** with I_2_ in pyridine (75 °C/15 min) gave cyclic compound **21** as its triethylammonium salts in a yield of 81%–87%. Finally, the removal of the TBDMS group of **21** was carried out in a solution of 1M TBAF/THF at room temperature for 1 h to obtain the target compound **5**. Compound **5** was identified by ^1^H- and ^31^P-NMR, and HRMS. 

### 2.2. Pharmacology

**Figure 2:**
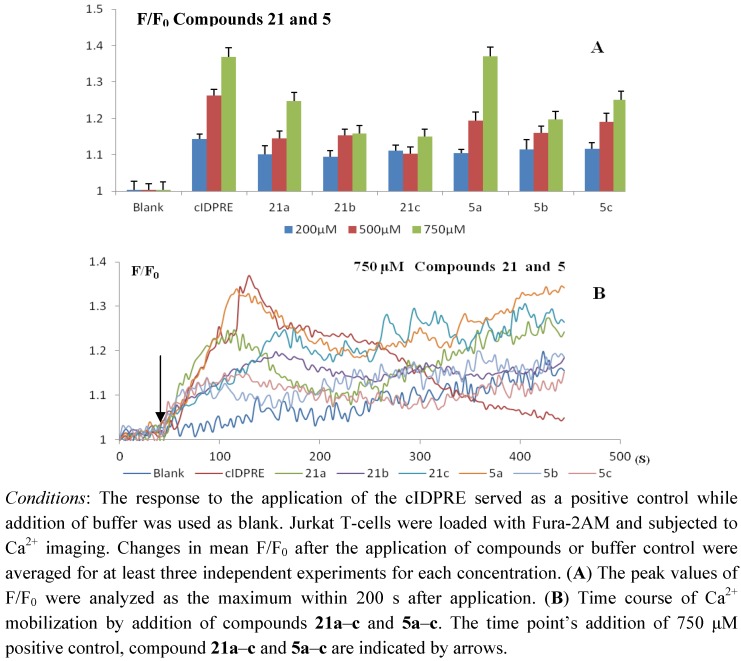
Ca^2+^ mobilization activities of compounds **21** and **5** in intact Jurkat T cells.

The Ca^2+^-mobilizing ability of the newly synthesized cADPR analogues **5a**–**c** were evaluated in Jurket-T cells. For studying the effect of the 3'-OH on activity, the 3'-*O*-TBDMS substituted precursors **21a**–**c** were also studied. The results are shown in Figure 2.

In general, the synthesized cADPR analogues are membrane-permeating agonists of the calcium signaling pathway. All compounds showed typical biphasic Ca^2+^ mobilizing kinetics with an initial immediate Ca^2+^ peak and a subsequent plateau phase. The induction of the immediate peak was strongest for compound **5a** at 750 μM, which was almost as active as the positive control cIDPRE, while for the other 2'-deoxy cADPR analogues a less pronounced initial Ca^2+^ peak was observed. The result indicates that the introduction of chlorine (**5b**) or fluorine (**5c**) to the 2'-deoxy position will lead to a decrease of activity.

In comparison to cIDPRE, however, 2'-deoxy cADPR analogues **21a**–**c** and **5a**–**c** sustained longer Ca^2+^ mobilization (Figure 2B), which implied their different molecular mechanism. 2'-Deoxy-cADPR was found to be inactive in Jurkat T cells [32]. Potter’s finding implied that the 2'-OH group is an important motif for the antagonistic activities of 8-substituted cADPR analogues and 2'-OH deletion may also result in an increase in the agonistic effects of the 8-substituted cADPR analogues [23,24]. This study shows that the agonistic activity is maintained in the case of disappearance of the 2'-OH, which emphasizes the importance of the structure of the nucleobase on the effect of the 2'-OH on calcium mobilization. 

The existence of a large hydrophobic *tert*-butyldimethylsilyl (TBDMS) group on the 3'-OH of the southern ribose (compounds **21a**–**c**) lowers activity, but most of the agonistic properties are still maintained. Potter *et al*. reported that the 3'-hydroxyl group is essential for Ca^2+^ releasing activity of cADPR in sea urchin eggs [23]. In our case, TBDMS attached compound **21** did not much alter the agonistic activity of compound **5**. This result may come from the existence of a hydrophobic group on the 3'-OH that makes compound more membrane permeant and partially compensates the negative effect. 

**Figure 3:**
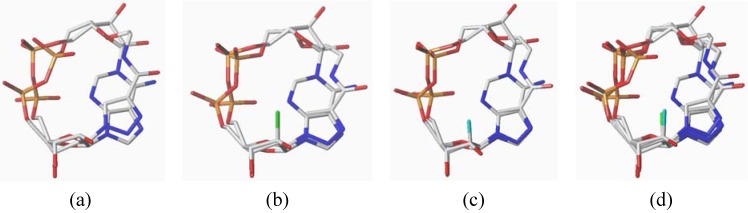
Superimposition of minimized conformations of cADPR and compounds **5a** (a), **5b** (b), **5c** (c) and **5a**–**c** (d).

The conformations of the modified cADPR analogues were investigated by molecular modeling. Their optimized conformations were superimposed with cADPR (Figure 3). The result clearly shows that **5a**–**c** and cADPR have very similar conformations. All riboses are in 2'-endo/3'-exo conformations and the whole backbones are in similar alignments. The consistence of conformations might result in the agonistic activities of cADPR analogues **5a**–**c**, even if the structures were simplified both in the southern ribose and nucleobase regions. For clearly understanding the structure-activity relationship, further detailed chemistry and/phamarcology investigations are needed.

## 3. Experimental

### 3.1. Chemistry

#### 3.1.1. General

HR-ESI-MS and ESI-MS were performed with a Bruker BIFLEX III instrument. ^1^H-NMR and ^13^C-NMR were recorded with a JEOL AL300 or a Bruker AVANCE III 400; CDCl_3_, D_2_O were used as solvents. Chemical shifts are reported in parts per million downfield from TMS (^1^H and ^13^C). ^31^P-NMR spectra (121.5 MHz) were recorded at room temperature by use of a JEOL AL300 spectrometer. Orthophosphoric acid (85%) was used as external standard. ^19^F-NMR spectra (470 MHz) were recorded on a Varian VXR-500 spectrometer. Chemical shifts of ^19^F-NMR are reported in ppm with reference to CF_3_COOH as external standard. Compounds were purified on an Alltech preparative C18 reversed-phase columns (2.2 × 25 cm) with a Gilson HPLC using MeCN/TEAB (pH 7.5) buffer system as eluent. Analytical TLC was performed using commercial glass plates coated to a thickness of 0.25 mm with Kieselgel 60 GF254 silica and visualized under UV light. Flash chromatography was performed using Qingdao (230–400 mesh) silica under a slight positive pressure of air. Microwave-assisted reactions were performed using a Biotage unit (400 w, 2.45 GHz). Solvents and regents for anhydrous reactions were dried prior to use by conventional methods.

#### 3.1.2. General Procedure for the Synthesis of **18a**–**c**

To a solution of compound **17** (185 mg, 0.44 mmol) and compound **16a**–**c** (0.44 mmol) in CH_3_CN (15 mL) was added CuI (10 mg, 0.05 mmol) and DIPEA (78 μL, 0.5 mmol), and the mixture was stirred at room temperature for 3 h. The mixture was evaporated *in vacuo* and the residue was partitioned between EtOAc and H2O. The aqueous phase was extracted again with EtOAc, then the organic layers were combined and dried (Na_2_SO_4_), filtered and concentrated *in vacuo*. The residue was purified by silica gel column chromatography (DCM-MeOH = 40:1–30:1) to afford a yellow oil. 

N*-(2-(2-*O*-Bis(phenylthio)phosphorylethoxy)ethyl)-1*H*-1*,*2*,*3-triazole-4-carboxamid-1-yl-2'-deoxy-3'-tert-butyldimethylsilyl-1'-*β*-D-ribofuranoside *(**18a**): Yield: 84%. ESI-TOF^+^: 695.2 [(M+H)^+^]. ^1^H-NMR (400 MHz, CDCl_3_): δ 8.50 (s, 1H, H-5), 7.54–7.37 (m, 10H, Ar-H), 7.42 (t, 1H, –NH–) 6.44 (dd, 1H, *J* = 8 Hz, H-1'), 4.49–4.47 (m, 1H, H-5'a), 4.25–4.23 (m, 1H, H-5'b), 4.34–4.30 (m, 2H, H-1"), 3.67–3.64 (m, 2H, H-3', H-4'), 3.62–3.56 (m, 4H, H-2", H-3"), 2.77–2.73 (m, 1H, H-2'a), 2.45 (dd, 1H, *J*_2'a,2'b_ = 16 Hz H-2'b), 0.82 (s, 9H, 3×CH_3_), 0.06 (s, 6H, 2×–Si(CH_3_)–). ^13^C-NMR (75 MHz, CDCl_3_): δ 160.3, 143.2, 135.7, 129.4, 126.2, 90.1, 89.5, 76.7, 70.1, 66.8, 62.0, 42.5, 39.1, 25.6, 17.9, −5.0. ^31^P-NMR (D_2_O, decoupled with ^1^H): *δ *50.91 (s). 

N*-(2-(2-*O*-Bis(phenylthio)phosphorylethoxy)ethyl)-1*H*-1*,*2*,*3-triazole-4-carboxamid-1-yl-2'-chloro-3'-tert-butyldimethylsilyl-1'-*β*-D-arabinoside* (**18b**): Yield: 85%. ESI-TOF^+^: 729.2 [(M+H)^+^]. ^1^H-NMR (400 MHz, CDCl_3_): δ 8.34 (s, 1H, H-5), 7.54–7.24 (m, 10H, Ar-H), 6.33 (dd, 1H, *J* = 8 Hz, H-1'), 4.73 (dd, 1H, *J*_5'a,5'b_ = 12Hz, H-5'a), 4.50 (dd, 1H, *J*_5'a,5'b_ = 12 Hz, H-5'b), 4.36–4.30 (m, 2H, H-4', H-3'), 4.41–3.90 (m, 2H, H-1"), 3.85–3.80 (m, 1H, H-2'), 3.68–3.61 (m, 2H, H-2"), 3.59–3.58 (m, 2H, H-4", H-3"), 0.89 (s, 9H, 3×CH_3_), 0.13 (s, 6H, 2×–Si(CH_3_)–). ^13^C-NMR (75 MHz, CDCl_3_): δ 160.2, 142.5, 135.3, 129.5, 126.3, 88.5, 77.3, 69.7, 66.8, 60.6, 38.6, 25.0, 17.8, −4.5. ^31^P-NMR (D_2_O, decoupled with ^1^H): *δ *51.27 (s).

N*-(2-(2-*O*-Bis(phenylthio)phosphorylethoxy)ethyl)-1*H*-1*,*2*,*3-triazole-4-carboxamid-1-yl-2'-fluoro-3'-tert-butyldimethylsilyl-1'-*β*-D-arabinoside* (**18c**): Yield: 83%. ESI-TOF^+^: 713.2 [(M+H)^+^]. ^1^H-NMR (400 MHz, CDCl_3_): δ 8.40 (s, 1H, H-5), 7.50–7.30 (m, 10H, Ar-H), 6.44 (dd, 1H, *J* = 8 Hz, H-1'), 5.08–4.93 (m, 1H, H-5'a) 4.65–4.59 (m, 1H, H-5'b), 4.30–4.28 (m, 2H, H-2', H-4'), 4.01–4.00 (m, 1H, H-3'), 3.78–3.54 (m, 8H, H-1'', H-4'', H-2'', H-3''), 0.86 (s, 9H, 3×CH_3_), 0.08 (s, 6H, 2×–Si(CH_3_)–).^13^C-NMR (75 MHz, CDCl_3_): δ 160.0, 143.1, 135.3, 129.4, 126.3, 96.2, 66.6, 60.7, 53.5, 38.7, 25.5, 17.8, 5.2. ^31^P-NMR (D_2_O, decoupled with ^1^H): *δ *51.09 (s).

#### 3.1.3. General Procedure for the Synthesis of **20a**–**c**

Compound **18a**–**c** (0.10 mmol) was dissolved in anhydrous CH_3_CN (5 mL). DIPEA (0.42 mmol) and POCl_3_ (0.35 mmol) were added successively to the solution at −20 °C. The mixture was stirred at 0 °C for 16 h, and then TEAB (5 mL, 1 M, pH 7.5) were added at 0 °C and the stirring was continued for 1 h at room temperature. After evaporation under reduced pressure, the residue was partitioned between H_2_O and CHCl_3_, and the aqueous layer was washed with CHCl_3_ and evaporated *in vacuo*. The residue was dissolved in TEAB buffer (5 mL, 0.05 M, pH 7.5), and applied to a C18 reversed-phase column (2.2 × 25 cm). The column was eluted using a linear gradient of 0–80% CH_3_CN in TEAB buffer (0.05 M, pH 7.5) to give **19a**–**c** as its triethylammonium salt. The residue was dissolved in 1 M TEAB (5 mL) and stirred at room temperature for 12 h, and then evaporated *in vacuo*. The residue was partitioned between H_2_O and CHCl_3_, and the aqueous layer was washed with CHCl_3_. Evaporated *in vacuo*, dissolved in TEAB buffer (1 mL, 0.05 M, pH 7.5), then applied to a C18 reversed-phase column (2.2 cm × 25 cm) eluted by a linear gradient of 0–80% CH_3_CN in TEAB buffer (0.05 M, pH 7.5) to give **20a**–**c** as triethylammonium salts.

N*-(2-(2-*O*-Phenylthiophosphorylethoxy)ethyl)-1*H*-1*,*2*,*3-triazole-4-carboxamid-1-yl-2'-deoxy-3'-tert-butyldimethylsilyl-5'-phosphoryl-1'-*β*-D-ribofuranoside *(**20a**): Yield: 54%. HRMS (ESI-TOF^−^) Calcd for C_24_H_40_N_4_O_11_P_2_SSi: [(M−H)^+^] 681.1586; Found: 681.1557. ^1^H-NMR (400 MHz, D_2_O): δ 8.47 (s, 1H, H-5), 7.47–7.17 (m, 5H, Ar-H) 6.42 (d, 1H, *J* = 8 Hz, H-1'), 4.48 (t, 1H, *J*_5'a,5'b_ = 4Hz, H-5'a), 4.41 (t, 1H, *J*_5'a,5'b_ = 4Hz, H-5'b), 3.98–3.96 (m, 2H, H-4', H-3'), 3.57–3.44 (m, 4H, H-2'',H-3''), 3.43–3.40 (m, 2H, H-4''), 3.07 (q, –NCH_2_–), 2.70–2.68 (m, 1H, H-2'a), 2.34 (d, 1H, H-2'b), 1.02 (t, –CH_3_–), 0.58 (s, 9H, 3×CH_3_), 0.07 (s, 6H, 2×–Si(CH_3_)–). ^31^P-NMR (D_2_O, decoupled with ^1^H): *δ *15.01 (br, s), 1.70 (br, s). 

N*-(2-(2-*O*-Phenylthiophosphorylethoxy)ethyl)-1*H*-1*,*2*,*3-triazole-4-carboxamid-1-yl-2'-chloro-3'-tert-butyldimethylsilyl-5'-phosphoryl-1'-*β*-D-arabinoside* (**20b**): Yield: 52%. HRMS (ESI-TOF^−^) Calcd for C_24_H_39_ClN_4_O_11_P_2_SSi: [(M−H)^+^] 715.1196; Found: 715.1186. ^1^H-NMR (400 MHz, D_2_O): δ 8.55 (s, 1H, H-5), 7.36–7.06 (m, 5H, Ar-H), 6.47–6.45 (m, 1H, H-1'), 4.48–4.46 (m, 2H, H-5'a, H-5'b), 4.59–4.03 (m, 2H, H-4', H-3'), 4.41–3.90 (m, 2H, H-1''), 3.96–3.95 (m, 1H, H-2'), 3.53–3.49 (m, 4H, H-2'', H-3''), 3.39–3.37 (m, 2H, H-4''), 3.02 (q, –NCH_2_–), 1.02 (t, –CH_3_–), 0.67 (s, 9H, 3×CH_3_), 0.1 (s, 6H). ^31^P-NMR (D_2_O, decoupled with ^1^H): *δ *18.24 (br, s), 1.16 (br, s).

N*-(2-(2-*O*-Phenylthiophosphorylethoxy)ethyl)-1*H*-1*,*2*,*3-triazole-4-carboxamid-1-yl-2'-fluoro-3'-tert-butyldimethylsilyl-5'-phosphoryl-1'-*β*-D-arabinoside* (**20c**): Yield: 50%. HRMS (ESI-TOF^−^) Calcd for C_24_H_39_FN_4_O_11_P_2_SSi: [(M−H)^+^] 699.1492; Found: 699.1511*. *^1^H-NMR (400 MHz, D_2_O): δ 8.46 (s, 1H, H-5), 7.36–7.09 (m, 5H, Ar-H), 6.50–6.46 (m, 1H, H-1'), 5.30–5.08 (m, 1H, H-4'), 4.11–4.10 (m, 1H, H-5'a), 3.98–3.96 (m, 1H, H-5'b), 3.94–3.91 (m, 3H, H-2', H-1''), 3.57–3.52 (m, 4H, H-2'',H-3''), 3.42–3.39 (m, 2H, H-4''), 3.02 (q, –NCH_2_–), 1.09 (t, –CH_3_–), 0.74 (s, 9H, 3×CH_3_), 0.01 (s, 6H, 2×–Si(CH_3_)–). ^31^P-NMR (D_2_O, decoupled with ^1^H): *δ *18.28 (br, s), 1.30 (br, s).

#### 3.1.4. General Procedure for the Synthesis of **21a**–**c**

The mixture of compound **20a**–**c**(12.4 mmol) and iodine (73 mg, 0.29 mmol) in pyridine (8 mL) was stirred at 75 °C, assisted by microwave irradiation, for 15 min. Then the pyridine was evaporated, and the residue was partitioned between CHCl_3_ and H_2_O. The aqueous layer was evaporated and the residue was dissolved in 0.05 M TEAB buffer (5.0 mL), and applied to a C18 reversed-phase column (2.2 × 25 cm). The column was eluted using a linear gradient of 0–80% CH_3_CN in TEAB buffer (0.05 M, pH 7.5) to give **21a**–**c** as triethylammonium salts.

N*-(2-(2-*O*-Phosphorylethoxy)ethyl)-1*H*-1*,*2*,*3-triazole-4-carboxamid-1-yl-2'-deoxy-3'-tert-butyldimethylsilyl-5'-phosphoryl-1'-*β*-D-ribofuranoside 2*,*5'-cyclicpyrophosphate *(**21a**): Yield: 87%. HRMS (ESI-TOF^−^) Calcd for C_18_H_34_N_4_O_11_P_2_Si: [(M−H)^+^] 571.1395; Found: 571.1380. ^1^H-NMR (400 MHz, D2O): δ 8.77 (s, 1H, H-5), 6.39–6.36 (m, 1H, H-1'), 4.47–4.46 (m, 1H, H-5'a), 4.42–4.41 (m, 1H, H-5'b), 4.35–4.34 (m, 2H, H-4', H-3'), 3.96–3.95 (m, 4H, H-2'', H-3''), 3.64–3.59 (m, 2H, H-4''), 3.04 (q, –NCH_2_–), 2.57–2.52 (m, 1H, H-2'a), 2.32–2.27 (m,1H, H-2'b), 1.12 (t, –CH_3_–), 0.52 (s, 9H, 3×CH_3_), 0.12 (s, 6H). ^31^P-NMR (D_2_O, decoupled with ^1^H): *δ *−11.69 (br, s), −12.01 (br, s).

N*-(2-(2-*O*-Phosphorylethoxy)ethyl)-1*H*-1*,*2*,*3-triazole-4-carboxamid-1-yl-2'-chloro-3'-tert-butyldimethylsilyl-5'-phosphoryl-1'-*β*-D-arabinoside 2*,*5'-cyclicpyrophosphate* (**21b**): Yield: 82%. HRMS (ESI-TOF^−^) Calcd for C_18_H_33_ClN_4_O_11_P_2_Si: [(M−H)^+^] 605.1006; Found: 605.1002. ^1^H-NMR (400 MHz, D_2_O): δ 9.13 (s, 1H, H-5), 6.60 (d, 1H, *J* = 8 Hz, H-1'), 4.76 (d, 1H, *J*_5'a,5'b_ = 8Hz, H-5'a), 4.55–4.53 (d, 1H, *J*_5'a,5'b_ = 8 Hz, H-5'b), 4.26–4.21 (m, 1H, H-4'), 4.14–4.13 (2H, m, H-1''), 4.05–4.01 (1H, m, H-3'), 3.91–3.88 (1H, m, H-2'), 3.72–3.64 (m, 4H, H-2'',H-3''), 3.58–3.35 (m, 2H, H-4''), 3.02 (q, –NCH_2_–), 1.16 (t, –CH_3_–), 0.79 (s, 9H, 3×CH_3_), 0.09 (s, 6H, 2×–Si(CH_3_)–). ^31^P-NMR (D_2_O, decoupled with ^1^H): *δ *−12.93 (br, s), −14.43 (br, s).

N*-(2-(2-*O*-Phosphorylethoxy)ethyl)-1*H*-1*,*2*,*3-triazole-4-carboxamid-1-yl-2'-fluoro-3'-tert-butyldimethylsilyl-5'-phosphoryl-1'-*β*-D-arabinoside 2*,*5'-cyclicpyrophosphate* (**21c**): Yield: 81%. HRMS (ESI-TOF^−^) Calcd for C_18_H_33_FN_4_O_11_P_2_Si: [(M−H)^+^] 589.1302; Found: 589.1325. ^1^H-NMR (400 MHz, D_2_O): δ 9.09 (s, 1H, H-5), 6.63 (dd, 1H, *J* = 8 Hz, H-1'), 5.36–5.21 (m, 1H, H-4'), 4.20–4.16 (m, 1H, H'-5a), 4.16–4.14 (m, 1H, H-5'b), 4.10–3.91 (m, 3H, H-2', H-1''), 3.68–3.52 (m, 4H, H-2'', H-3''), 3.54–3.34 (m, 2H, H-4''), 3.01 (q, –NCH_2_–), 1.13 (t, –CH_3_–), 0.79 (s, 9H, 3×CH_3_), 0.09 (s, 6H, 2×–Si(CH_3_)–). ^31^P-NMR (D_2_O, decoupled with ^1^H): *δ *−12.90 (br, s), −14.22 (br, s).

#### 3.1.5. General Procedure for the Synthesis of **5a**–**c**

Compound **21a**–**c** (33.6 μmol) was dissolved in 1M TBAF/THF (0.5 mL) and the solution was stirred for 2 h, and then was evaporated under reduced pressure. The residue was dissolved in 0.05 M TEAB buffer (2.0 mL), which was loaded to C18 reversed-phase column (2.2 × 25 cm). The column was eluted using a linear gradient of 0–80% CH_3_CN in TEAB buffer (0.05 M, pH 7.5) to afford **5a**–**c** as triethylammonium salts.

N*-(2-(2-*O*-Phosphorylethoxy)ethyl)-1*H*-1*,*2*,*3-triazole-4-carboxamid-1-yl-2'-deoxy-5'-phosphoryl-1'-*β*-D-ribofuranoside 2*,*5'-cyclicpyrophosphate* (**5a**): Yield: 36%. HRMS (ESI-TOF^−^) Calcd for C_12_H_20_N_4_O_11_P_2_: [(M+H)^+^] 459.1051; Found: 459.1040. ^1^H-NMR (400 MHz, D_2_O): δ 8.47 (s, 1H, H-5), 6.48–6.42 (m, 1H, H-1'), 4.48–4.47 (m, 1H, H'-5a), 4.37–4.30 (m, 1H, H-5'b), 3.99–3.97 (m, 2H, H-1''), 3.87–3.86 (m, 1H, H-3'), 3.61–3.55 (m, 4H, H-3'', H-4''), 3.46–3.43 (m, 2H, H-2''), 3.09 (q, –NCH_2_–), 2.89–2.77 (m, 1H, H-2'a), 2.43–2.40 (m, 1H, H-2'b), 1.17 (t, –CH_3_–). ^31^P-NMR (D_2_O, decoupled with ^1^H), *δ* *−*9.77 (br, s), −10.05 (br, s).

N*-(2-(2-*O*-Phosphorylethoxy)ethyl)-1*H*-1*,*2*,*3-triazole-4-carboxamid-1-yl-2'-chloro-5'-phosphoryl-1'-*β*-D-arabinoside 2*,*5'-cyclicpyrophosphate *(**5b**): Yield: 31%. HRMS (ESI-TOF^−^) Calcd for C_12_H_19_ClN_4_O_11_P_2_: [(M−H)^+^] 491.0141; Found: 491.0142. ^1^H-NMR (400 MHz, D_2_O): δ 9.17 (s, 1H, H-5), 6.60 (d, 1H, *J* = 8 Hz, H-1'), 4.77–4.73 (m, 2H, H-5'a, H-5'b), 4.26–4.21 (m, 1H, H-4'), 4.24–4.09 (2H, m, H-1''), 4.00–3.98 (1H, m, H-3'), 3.69–3.66 (1H, m, H-2'), 3.50–3.40 (m, 4H, H-2'', H-3''), 3.10–3.04 (m, 2H, H-4''). 3.04 (q, –NCH_2_–), 1.15 (t, –CH_3_–). ^31^P-NMR (D_2_O, decoupled with ^1^H): *δ* −7.58 (br, s), −9.00 (br, s).

N*-(2-(2-*O*-Phosphorylethoxy)ethyl)-1*H*-1*,*2*,*3-triazole-4-carboxamid-1-yl-2'-fluoro-5'-phosphoryl-1'-*β*-D-arabinoside 2*,*5'-cyclicpyrophosphate* (**5c**): Yield: 29%. HRMS (ESI-TOF^−^) Calcd for C_12_H_19_FN_4_O_11_P_2_: [(M−H)^+^] 475.0437; Found: 475.0449. ^1^H-NMR (400 MHz, D_2_O): δ 9.14 (s, 1H, H-5), 6.64 (m, 1H, H-1'), 5.46–5.43 (m, 1H, H-5'a), 5.33–5.32 (m, 1H, H-5'b), 4.20–4.15 (m, 2H, H-1''), 4.02–3.99 (m, 2H, H-2', H-4'), 3.69–3.67 (m, 2H, H-2''), 3.52–3.41 (m, 4H, H-3'', H-4''), 3.06 (q, –NCH_2_–), 2.96–2.94 (m, 1H, H-3'), 1.13 (t, –CH_3_–). ^31^P-NMR (D_2_O, decoupled with ^1^H): *δ* −9.57 (br, s), −10.53 (br, s). ^19^F-NMR (D_2_O, CF_3_COOH), *δ*-202.13–202.32 (m).

### 3.2. Biological Studies

Cell Culture. The human Jurkat T-lymphocyte cell line was obtained from the Hongkong University Physiology Department Professor Lee Hon Cheung’s lab Culture Collection. It was cultured in RPMI Medium 1640 (Invitrogen, Carlsbad, CA, USA) supplemented with 10% fetal bovine serum (FBS), 100 units/mL penicillin, and 2% Hepes Buffer (1 M, pH 7.4) in a humidified (5% CO_2_) atmosphere at 37 °C.

Calcium agonistic activity measurement. The cells (3 × 10^5^ cells/well) were plated in 24-well plate with poly L-lysine (100 μg/mL) and incubated in no FBS and penicillin medium at 37 °C overnight for adherence. Before measurement, cells were incubated with Fura-2 AM (2 μM) in HBSS for 20 min in dark at 37 °C. Then cells were washed with calcium measurement buffer HBSS twice and added 200 μL HBSS for measurement. Changes in Fura-2 fluorescence were measured using an Olympus Cell^R^ Live-cell confocal imaging system, operating in ratio mode (alternating excitation at 340 and 380 nm). Each compound was dissolved at tested concentrations in 50 μL HBSS and added into the cells at a time point 40 s after the start of each measurement. Data (F: ratio between emission at 340 and 380 nm) were recorded for the next 500 s. Changes in mean F/F_0_ (F_0_: Ratio between emission at 340 and 380 nm at the start point of measurement) after the application of compounds were averaged for at least three independent experiments for each concentration. The peak values of F/F_0_ were analyzed as the maximum within 200 s after compound application, shown in the ordinate of Figure 2. Response to the application of cIDPRE was served as a positive control while addition of buffer was used as blank.

### 3.3. Calculations

The conformations of studied cADPR analogues were optimized with density functional theory (DFT) quantum chemical method by using Gaussian 09 program package [33]. Equilibrium geometries of all molecules were fully optimized at the B3LYP/6–31G(d) level of theory [34]. Vibrational frequencies, calculated at the same levels, were used to determine the nature of the stationary points and to give the thermodynamic date and zero-point vibrational energies. Then SYBYL-X 1.1 software was used to align the calculated structures of three cADPR analogs with crystal structure of cADPR, respectively.

## 4. Conclusions

In summary, novel nucleobase-modified and sugar-modified cADPR mimics **5** were synthesized, in which halogen atoms were introduced into the southern ribose for the first time. The synthesis employed a Cu(I)-catalyzed Hüisgen [3+2] cycloaddition for the building of the triazole moiety and microwave-assisted reactions were used for the pyrophosphate bond formation. Biological evaluation reveals that these new kinds of cADPR analogues are membrane permeant agonists of the calcium signaling pathway. The introduction of chlorine or fluorine into the 2'-position of the southern ribose led to a decrease of activity. The existence of a hydrophobic group on the 3'-OH of the southern ribose does not alter much the agonistic activity. The result of this study is helpful for the understanding of structure-activity relationships of cADPR analogues and to help design new probes to investigate the cADPR-mediated calcium signaling pathway. 
